# Musculoskeletal ultrasonography in routine rheumatology practice: data from Central and Eastern European countries

**DOI:** 10.1007/s00296-016-3442-2

**Published:** 2016-02-29

**Authors:** Peter Mandl, Asta Baranauskaite, Nemanja Damjanov, Maja Hojnik, Reka Kurucz, Orsolya Nagy, Petr Nemec, Dora Niedermayer, Porin Perić, Tzvetanka Petranova, Andres Pille, Simona Rednic, Violeta Vlad, Martin Zlnay, Peter V. Balint

**Affiliations:** Division of Rheumatology, 3rd Department of Internal Medicine, Medical University of Vienna, 18-20 Währinger Gürtel, Vienna, Austria; 3rd Department of Rheumatology, National Institute of Rheumatology and Physiotherapy, Budapest, Hungary; Department of Rheumatology, Lithuanian University of Health Sciences, Kaunas, Lithuania; Institute of Rheumatology, University of Belgrade School of Medicine, Belgrade, Serbia; Global Medical Affairs Rheumatology, AbbVie, Ljubljana, Slovenia; AbbVie Ltd., Budapest, Hungary; Department of Rheumatology, St. Anne’s University Hospital, Brno, Czech Republic; Department of Rheumatology, Clinical Hospital Center Zagreb, University of Zagreb, Zagreb, Croatia; Department of Rheumatology, UMHAT St.Iv.Rilsky, Medical University, Sofia, Bulgaria; East-Tallinn Central Hospital, Tallinn, Estonia; Department of Rheumatology, Clinical County Emergency Hospital, Cluj-Napoca, Romania; Department of Rheumatology, Sf. Maria Clinical Hospital, Bucharest, Romania; Department of Rheumatology, National Institute of Rheumatic Diseases, Piestany, Slovak Republic

**Keywords:** Ultrasonography, Musculoskeletal, Central-Eastern Europe, Clinical practice, Education

## Abstract

The main aim was to gain structured insight into the use of musculoskeletal ultrasonography (MSUS) in routine rheumatology practices in Central and Eastern European (CEE) countries. In a cross-sectional, observational, international, multicenter survey, a questionnaire was sent to investigational sites in CEE countries. Data on all subsequent routine MSUS examinations, site characteristics, MSUS equipment, and investigators were collected over 6 months or up to 100 examinations per center. A total of 95 physicians at 44 sites in 9 countries provided information on a total of 2810 MSUS examinations. The most frequent diagnoses were rheumatoid arthritis (RA) and spondyloarthritis (34.8 and 14.9 % of cases, respectively). Mean number of joints examined was 6.8. MSUS was most frequently performed for diagnostic purposes (58 %), particularly in patients with undifferentiated arthritis, suspected soft tissue disorders, or osteoarthritis (73.0–85.3 %). In RA patients, 56.3 % of examinations were conducted to monitor disease activity. Nearly all investigations (99 %) had clinical implications, while the results of 78.6 % of examinations (51.6–99.0 %) were deemed useful for patient education. This first standardized multicountry survey performed in CEEs provided a structured documentation of the routine MSUS use in participating countries. The majority of MSUS examinations were performed for diagnostic purposes, whereas one-third was conducted to monitor disease activity in RA. A majority of examinations had an impact on clinical decision making and were also found to be useful for patient education.

**Electronic supplementary material:**

The online version of this article (doi:10.1007/s00296-016-3442-2) contains supplementary material, which is available to authorized users.

## Introduction

In the past decade, musculoskeletal ultrasonography (MSUS) has been extensively used by an ever-growing number of rheumatologists in both research studies as well as in daily clinical practice. The need to monitor individual patients, along with the recognition that MSUS can depict subclinical synovitis and enthesitis, have been the main drivers behind the escalating utilization of MSUS [1–4]. In addition, MSUS has the potential to reliably guide treatment interventions (e.g., needle aspiration, intra-articular injections) [5, 6] and was shown to have profound effects on disease classification and physician decision making [7, 8]. Progress in practice was accompanied by an increasing need for organized education regarding the use of MSUS [6, 9, 10]. European guidelines for education and documentation of MSUS for rheumatologists, including those issued by the European Federation of Societies of Ultrasound in Medicine and Biology were issued [10–12]. Advancements in research and education were accompanied by increasing access to MSUS devices among rheumatologists [13]. Nowadays, an increasing number of hospital departments, outpatient clinics, and emergency departments equip their facilities with ultrasound devices suitable for MSUS imaging.

Most of the available data on MSUS in the field of rheumatology has been collected within the framework of clinical studies. Data on the actual usage and utilization of MSUS equipment in routine clinical practice by rheumatologists in Europe, however, are limited and outdated. The few available surveys collected information primarily on the expertise and training of the investigators, rather than on the MSUS examinations themselves [6, 9, 14]. Detailed analysis of such examinations would be particularly relevant for improving MSUS utilization and value in general and could facilitate ongoing international research activities. Such data are particularly lacking for Central and Eastern Europe, a region in which we know almost nothing about the overall acceptance of MSUS in rheumatologic practice.

The primary objective of the Musculoskeletal UltraSound as a Tool (MUST) study was to gain insight into the use of MSUS imaging in routine rheumatologic care in Central and Eastern European (CEE) countries.

## Methods

This cross-sectional, observational, multicenter survey was conducted in the following nine CEE countries: Bulgaria, Croatia, the Czech Republic, Estonia, Hungary, Lithuania, Romania, the Republic of Serbia, and the Slovak Republic. From the remaining countries in the CEE region, representatives from Albania, Latvia, Poland, and Slovenia were contacted by the principal investigators; however, these countries declined to participate in the study. Montenegro, Macedonia, Kosovo, and Bosnia–Herzegovina were omitted because of the absence of country representation with regard to the study sponsor (AbbVie).

Investigational sites were selected based on the availability of MSUS equipment at the site and were identified with help from the national societies to ensure representative coverage in each individual country. The aim was to collect approximately 3000 data sets from approximately 40–50 sites.

Participating sites recorded information on the methodology of all consecutive MSUS examinations performed over a 6-month period or up to 100 ultrasonography events, regardless of diagnosis, extent, location, or purpose of MSUS imaging. Data for site and investigator characteristics were recorded by using purpose-designed case report forms (CRFs) in English. Additional CRFs were used for capturing information about the individual MSUS examinations.

The following endpoints were investigated: frequency of MSUS imaging at study sites; the percentage of MSUS examinations performed at various stages of individual disorders; the purpose of MSUS imaging; and the impact of MSUS findings on clinical decision making and on the professional conduct of the investigator. Furthermore, the study gathered information on the study site, ultrasound equipment, number of rheumatologists, and their level of competence in diagnostic MSUS, as well as on patient turnover at individual sites.

For the statistical analysis, three analysis sets were utilized: (1) a site-based analysis set (SBS), which included all data on participating study sites documenting at least 1 MSUS investigation; (2) an investigator-based analysis set (IBS), including all data on MSUS investigators at participating study sites (i.e., those included in the SBS); and (3) an examination-based analysis set (EBS), comprised of data on all documented MSUS investigations.

The study was approved by the appropriate national ethics committees in each participating CEE country. Study-related activities started only after informed consent was obtained from the patients.

The statistical analyses were carried out with version 9.2 of Statistical Analysis System software (SAS Institute Inc., Cary, NC, USA). Data were stratified according to study site, investigator, and type of examination. Simple descriptive and summary statistics were calculated. Qualitative data (e.g., gender) were generally presented as frequency distributions. For the calculation of percentages, missing data (denoting data not entered into the CRF) were generally considered as a separate group. Quantitative data (e.g., age) were analyzed by statistical parameters such as valid *N*, mean, standard deviation (SD), minimum, maximum, and median. Where applicable and interpretable, 95 % confidence intervals (CIs) are provided. All analyses were generally performed overall and by country.

## Results

### MSUS sites (SBS analysis)

Data were collected on 2810 MSUS examinations performed by 95 rheumatologists at 44 study sites in nine CEE countries, during the observation period from July 2011 to October 2012. The number of sites included for each country was as follows: Romania, ten sites; Bulgaria, eight sites; Hungary and Czech Republic, six sites each; Slovakia, four sites; Estonia and Croatia, three sites each; and Lithuania and Serbia, two sites each.

The majority of sites were either university hospitals (25 sites; 56.8 %) or other hospitals (11 sites; 25.0 %), while seven additional centers included private practices (4 sites; 9.1 %), outpatient clinics (1 site; 2.3 %), and other medical institutions (2 sites; 4.5 %). These data were missing for a single site (2.3 %).

Annual turnover of rheumatology patients undergoing MSUS was between 1000 and 10,000 in the majority (68.2 %) of study sites. The largest data sets were reported from Hungarian (546 MSUS examinations), Romanian (500 MSUS examinations), and Estonian (351 MSUS examinations) sites. A mean of 4.6 ± 3.3 (SD) MSUS examinations were carried out weekly at these sites, with the lowest mean of 1.5 ± 1.6 examinations in Bulgaria and the highest mean of 9.0 ± 3.6 examinations in Lithuania.

Over all study sites, on average, 3.7 ± 2.6 physicians were trained in the application of MSUS. The mean number of trained rheumatologists was highest in Serbia (6.5 ± 2.1) and lowest in Slovakia (2.0 ± 0.8). The majority of the rheumatologist experts undertook either any one of the official EULAR MSUS courses (beginner, intermediate or advanced) or EULAR-endorsed MSUS courses, while a smaller number undertook national courses.

The brand and type of MSUS equipment varied between (and in some cases within) each CEE country. Information on the major brands and type of MSUS equipment within the CEE region, along with the transducer frequencies, is provided in Supplementary Table 1.

### MSUS investigators (IBS analysis)

MSUS investigators carried out a mean number of 29.6 ± 18.5 MSUS weekly examinations, ranging from a mean of 8.7 ± 3.4 examinations in Bulgaria to up to 60.0 ± 22.4 examinations in Serbia. The mean number of MSUS courses attended by the rheumatologist investigators was 2.9 ± 2.3. On average, physicians in the Serbian study sites attended the highest number of MSUS courses, with a mean number of 8.3 ± 4.6, whereas physicians in Lithuania attended only 1.5 ± 0.6 courses. Every second physician (*n* = 49; 51.6 %) attended at least one advanced level MSUS course, while 39 (41.1 %) attended at least one basic level course.

### MSUS examinations (EBS analysis)

#### Scheduling of the MSUS examinations

Most MSUS examinations (51.9 %) were scheduled ad hoc, whereas 43.4 % of examinations were elective. While the rate of ad hoc investigations was highest in Lithuania (76.3 %) and lowest in Bulgaria (20.4 %), elective examinations were most frequent in Croatia (65.0 %) and least frequent in Lithuania (17.7 %). The majority of examinations were initial investigations (79.6 %), whereas only 18.3 % were repeated. In Hungary, the rate of initial examinations was highest at 88.5 % (and lowest with 9.9 % of repeated investigations); in Bulgaria, the rates were 55.8 % (initial) and 42.5 % (repeated).

#### Diseases assessed with the MSUS examinations

The most common diagnosis within this study was rheumatoid arthritis (RA), which was diagnosed in 34.8 % of the examined patients (Table 1). Other common diagnoses included spondyloarthritis (SpA; 14.9 %), soft tissue disorders (14.7 %), osteoarthritis (OA; 12.9 %) and undifferentiated arthritis (UA; 10.9 %); all other diagnoses [juvenile idiopathic arthritis, gout, calcium pyrophosphate dehydrate (CPPD) deposition disease, septic arthritis, and systemic lupus erythematosus] represented <10 % of patients. Nearly every second patient (51.9 %) was classified as having chronic disease at the time of the MSUS examination.

**Table 1:** MSUS examinations grouped by the most common diagnoses

Country	RA		SpA		Soft tissue disorder		OA		UA	
	*n*	%	*n*	%	*n*	%	*n*	%	*n*	%
Bulgaria	48	42.5	37	32.7	8	7.1	5	4.4	1	0.9
Czech Republic	75	37.5	45	22.5	20	10.0	18	9.0	25	12.5
Croatia	105	35.0	41	13.6	56	18.7	24	8.0	42	14.0
Estonia	94	26.8	72	20.5	58	16.5	47	13.4	35	10.0
Hungary	201	36.8	64	12.3	91	16.7	65	11.9	60	11.0
Lithuania	82	27.3	38	12.6	31	10.3	17	5.7	67	22.3
Romania	212	42.4	63	12.6	80	16.0	57	11.4	37	7.4
Serbia	54	18.0	15	5.1	64	21.3	113	37.7	30	10.0
Slovakia	107	53.5	41	20.5	6	3.0	17	8.5	8	4.0
Total	978	34.8	419	14.9	414	14.7	363	12.9	305	10.9

Country	Gout		JIA		CPPD		SA		SLE	
	*n*	%	*n*	%	*n*	%	*n*	%	*n*	%
Bulgaria	9	8.0	0	0.0	5	4.4	0	0.0	0	0.0
Czech Republic	7	3.5	6	3.0	0	0.0	0	0.0	1	0.5
Croatia	9	3.0	3	1.0	3	1.0	1	0.3	6	2.0
Estonia	16	4.6	11	3.1	6	1.7	0	0.0	3	0.9
Hungary	11	2.0	26	4.8	10	1.8	4	0.7	5	0.9
Lithuania	19	6.3	8	2.7	3	1.0	16	5.3	3	1.0
Romania	33	6.6	2	0.4	10	2.0	5	1.0	7	1.4
Serbia	9	3.0	0	0.0	3	1.0	0	0.0	0	0.0
Slovakia	2	1.0	4	4.0	1	0.5	2	1.0	3	1.5
Total	115	4.1	60	2.1	41	1.5	28	1.0	28	1.0

*n* number of MSUS examinations, *%* percentage of MSUS examinations by the individual diagnoses, *CPPD* calcium pyrophosphate dehydrate deposition disease, *JIA* juvenile idiopathic arthritis, *OA* osteoarthritis, *MSUS* musculoskeletal ultrasonography, *RA* rheumatoid arthritis, *SA* septic arthritis, *SLE* systemic lupus erythematosus, *SpA* spondyloarthritis, *UA* undifferentiated arthritis

#### Content of the MSUS examinations

Investigated structures were mostly joints (93.3 %); in 15.1 % of cases, entheses were examined, while the examination included other soft tissue structures in 23.0 % of examinations. The percentage of examinations investigating joints was relatively homogeneous between the different CEE countries (88.6–98.7 %); however, entheses were examined in only 6.8 % of MSUS examinations in Hungary, while 37.2 % were examined in Bulgaria.

The number of joints investigated per MSUS examination ranged between 1 and 46 joints, with a mean of 6.8 ± 8.4 joints. The highest mean number of investigated joints was reported in patients with RA (9.8 ± 9.4), followed by patients with unknown or missing diagnosis (9.4 ± 10.3). The fewest number of joints was reported for septic arthritis (1.3 ± 0.5). Similarly, the number of entheses ranged from 1 to 40, with a mean of 3.6 ± 3.5 entheses.

#### Purpose of the MSUS examinations

The majority of MSUS examinations (58.8 %) had a diagnostic purpose, while 33.2 and 15.0 % of examinations were performed to monitor disease activity or the efficacy of treatment, respectively. Guidance of an interventional procedure served as a purpose in 11.6 % of MSUS examinations. The highest rate of diagnostic investigations occurred in Lithuania (74.7 %), whereas the lowest occurred in Bulgaria (47.8 %). The proportion of examinations monitoring disease activity ranged from 9.3 % in Serbia to 49.4 % in Romania. The purpose of MSUS for monitoring treatment efficacy ranged from 7.3 % in Croatia to 35.4 % in Bulgaria and the use of MSUS to aid in an interventional procedure ranged from 0.3 % in Croatia to 22.8 % in Estonia. In 0.4 % of cases, the respective information was missing.

The purpose of the MSUS examinations differed between the different rheumatic diseases. While the majority of MSUS examinations (56.3 %) in patients with RA were performed for the purpose of monitoring disease activity, in patients with UA, as well as in those with suspected soft tissue disorders or OA, the MSUS examination primarily served a diagnostic purpose (82.0, 85.3, and 73.0 %, respectively; Table 2). In patients with SpA, the ratio between diagnostic and monitoring examinations was balanced (46.3 and 42.0 %, respectively).

**Table 2:** Purpose of MSUS examinations grouped by the most common diagnoses

Diagnosis	Diagnostic		Monitoring disease activity		Monitoring therapeutic efficacy		Procedure guidance	
	*n*	%	*n*	%	*n*	%	*n*	%
RA	356	36.4	551	56.3	252	25.8	85	8.7
SpA	194	46.3	176	42.0	79	18.9	51	12.2
Soft tissue disorders	353	85.3	35	8.5	23	5.6	64	15.5
OA	265	73.0	43	11.8	24	6.6	81	22.3
UA	250	82.0	44	14.4	16	5.2	24	7.9

Multiple choice was possible

*n* number of MSUS examinations, *%* percentage of MSUS examinations by the individual diagnoses, *MSUS* musculoskeletal ultrasonography, *OA* osteoarthritis, *RA* rheumatoid arthritis, *SpA* spondyloarthritis, *UA* undifferentiated arthritis

#### Impact of the MSUS examinations

In almost all cases, the MSUS examinations had at least some impact, with no impact implicated in only 28 cases (1.0 %). In 58.6 % of cases, the results of the examination confirmed a clinical suspicion; in 24.9 % of cases, it clarified an unclear pathology; in 23.6 % of cases, the examination resulted in change in treatment; in 14.7 % of cases, it revealed information that was different from the clinical investigation; and in 10.1 % of cases, it helped established a new diagnosis (Table 3).

**Table 3:** Impact of MSUS examinations

Country	Confirmed clinical findings		Differed From clinical findings		Established new diagnosis		Changed diagnosis		Clarified unclear pathology		Detected subclinical disease activity	
	*n*	%	*n*	%	*n*	%	*n*	%	*n*	%	*n*	%
Bulgaria	79	69.9	9	8.0	9	8.0	5	4.4	35	31.0	17	15.0
Czech Republic	116	58.0	34	17.0	20	10.0	3	1.5	34	17.0	18	9.0
Croatia	147	49.0	49	16.3	47	15.7	16	5.3	120	40.0	18	6.0
Estonia	196	55.8	59	16.8	28	8.0	9	2.6	28	8.0	9	2.6
Hungary	349	63.9	50	9.2	32	5.9	7	1.3	117	21.4	34	6.2
Lithuania	117	39.0	49	16.3	22	7.3	14	4.7	96	32.0	19	6.3
Romania	304	60.8	78	15.6	80	16.0	7	1.4	169	33.8	55	11.0
Serbia	196	65.3	70	23.3	34	11.3	45	15.0	58	19.3	5	1.7
Slovakia	144	72.0	16	8.0	13	6.5	4	2.0	43	21.5	22	11.0
Total	1648	58.6	414	14.7	285	10.1	110	3.9	700	24.9	197	7.0

Country	Changed clinical evaluation		Ended therapy		Changed therapy		Suggested further diagnostic evaluation		Other impact		No impact	
	*n*	%	*n*	%	*n*	%	*n*	%	*n*	%	*n*	%
Bulgaria	35	31.0	9	8.0	65	57.5	6	5.3	7	6.2	0	0.0
Czech Republic	7	3.5	2	1.0	42	21.0	4	2.0	14	7.0	1	0.5
Croatia	22	7.3	17	5.7	53	17.7	16	5.3	2	0.7	3	1.0
Estonia	17	4.8	12	3.4	71	20.2	8	2.3	53	15.1	5	1.4
Hungary	42	7.7	5	0.9	104	19.0	30	5.5	23	4.2	3	0.5
Lithuania	20	6.7	22	7.3	84	28.0	30	10.0	3	1.0	2	0.7
Romania	75	15.0	8	1.6	141	28.2	19	3.8	41	8.2	7	1.4
Serbia	10	3.3	14	4.7	61	20.3	10	3.3	3	1.0	5	1.7
Slovakia	16	8.0	2	1.0	43	21.5	10	5.0	0	0.0	2	1.0
Total	244	8.7	91	3.2	664	23.6	133	4.7	146	5.2	28	1.0

Multiple choice was possible

*n* number of US examinations, *%* percentage of US examinations within the respective country, *MSUS* musculoskeletal ultrasonography

Based on their reports, overall, the investigators considered the MSUS examination to be a reasonable use of their time in the vast majority of cases (96.6 %; Table 4). Investigators in Serbia showed considerably divergent assessments from the other countries in this context, with only 83.3 % of examinations classified as reasonable.

**Table 4:** MSUS as a reasonable use of time

Country	Reasonable		Not reasonable		Missing		Total	
	*n*	%	*n*	%	*n*	%	*n*	%
Bulgaria	112	99.1	0	0.0	1	0.9	113	100.0
Czech Republic	200	100.0	0	0.0	0	0.0	200	100.0
Croatia	290	96.7	9	3.0	1	0.3	300	100.0
Estonia	325	92.6	8	2.3	18	5.1	351	100.0
Hungary	542	99.3	3	0.5	1	0.2	546	100.0
Lithuania	298	99.3	0	0.0	2	0.7	300	100.0
Romania	498	99.6	2	0.4	0	0.0	500	100.0
Serbia	250	83.3	50	16.7	0	0.0	300	100.0
Slovakia	199	99.5	1	0.5	0	0.0	200	100.0
Total	2714	96.6	73	2.6	23	0.8	2810	100.0

*n* number of US examinations, *%* percentage of US examinations within the respective country, *MSUS* musculoskeletal ultrasonography

The results of 87.0 % of MSUS examinations were found to have educational value for the physician, while the results of only 11.8 % of examinations had no educational value for the physician (Table 5). Again, the lowest rate of positive assessment in this regard was observed in Serbia, with an educational value attributed to only 67.3 % of investigations.

**Table 5:** General educational effect of MSUS

Country	Educational value for physician				Missing data		Total	
	Yes		No					
	*n*	%	*n*	%	*n*	%	*n*	%
Bulgaria	101	89.4	9	8.0	3	2.7	113	100.0
Czech Republic	184	92.0	16	8.0	0	0.0	200	100.0
Croatia	292	97.3	8	2.7	0	0.0	300	100.0
Estonia	254	72.4	79	22.5	18	5.1	351	100.0
Hungary	469	85.9	76	13.9	1	0.2	546	100.0
Lithuania	278	92.7	12	4.0	10	3.3	300	100.0
Romania	468	93.6	32	6.4	0	0.0	500	100.0
Serbia	202	67.3	96	32.0	2	0.7	300	100.0
Slovakia	197	98.5	3	1.5	0	0.0	200	100.0
Total	2445	87.0	331	11.8	34	1.2	2810	100.0

*n* number of US examinations, *%*, percentage of US examinations within the respective country, *MSUS* musculoskeletal ultrasonography

Every third MSUS examination (33.3 %) was reported as not useful for teaching or sharing purposes (i.e., with colleagues), whereas in 65.1 % of cases, rheumatologists were able to utilize the results for either one of the aforementioned purposes. In 78.6 % of examinations, the results were used to educate the patient (Table 6). The highest and lowest rates (99.0 and 51.6 %) in this regard were observed in Slovakia and Estonia, respectively. In 2.5 % of examinations, information about the potential educational value for the patient was missing.

**Table 6:** The MSUS examination as a tool for patient education

Country	Educational purpose		Non-educational purpose		Missing data		Total	
	*n*	%	*n*	%	*n*	%	*n*	%
Bulgaria	103	91.2	9	8.0	1	0.9	113	100.0
Czech Republic	168	84.0	32	16.0	0	0.0	200	100.0
Croatia	287	95.7	13	4.3	0	0.0	300	100.0
Estonia	181	51.6	140	39.9	30	8.5	351	100.0
Hungary	406	74.4	140	25.6	0	0.0	546	100.0
Lithuania	216	72.0	46	15.3	38	12.7	300	100.0
Romania	420	84.0	80	16.0	0	0.0	500	100.0
Serbia	231	77.0	68	22.7	1	0.3	300	100.0
Slovakia	198	99.0	2	1.0	0	0.0	200	100.0
Total	2210	78.6	530	18.9	70	2.5	2810	100.0

*n* number of US examinations, *%*, percentage of US examinations within the respective country, *MSUS* musculoskeletal ultrasonography

## Discussion

This is the first study that provides comparative information about real-life MSUS examinations in routine rheumatologic care in CEE countries based on a structured questionnaire.

In recent years, MSUS has established itself as a highly sensitive and useful diagnostic tool for the detection of intra- and periarticular changes, including synovitis, tenosynovitis, bone erosions, and osteophytes, which are pivotal to most commonly occurring rheumatic diseases [15–19]. Reflecting this progress, MSUS is now a recommended imaging modality for diagnostic, monitoring, and predictive purposes with both RA and SpA [20, 21]. The data supporting such applications of MSUS in daily clinical practice are derived almost exclusively from observational studies. The vast majority of these studies, however, included selected patients, highly trained observers, as well as high-end ultrasound equipment. Information on the actual usage of MSUS in routine care is scarce to nonexistent, and was primarily assessed on a national level [22–28]. European surveys have reported considerable variation in training and practice between countries for both MSUS examinations in general and ultrasound-guided musculoskeletal interventions [6, 9, 14]. Rather than analyzing the standardized documentation of routine MSUS examinations, the available studies evaluated the needs and practice of participants (rheumatologists and/or rheumatologist experts in MSUS) [6, 9, 14, 22–29]. Therefore, it is difficult to draw comparisons between the findings of these studies and our study, which was based on the standardized documentation of routine MSUS examinations. Broadly speaking the main findings of our study are in accordance with other studies. In their study on the use of MSUS in the UK, Brown et al. [24] have found that the greatest clinical utility of MSUS were inflammatory arthritis assessment and guided procedures, findings which were also verified by Cunnington et al. [25]. Similarly to our study, a Romanian and a Spanish study could demonstrate that MSUS was primarily used as for diagnostic and monitoring purposes [27, 28].

Our survey, based on the standardized documentation of routine MSUS examinations, confirmed that MSUS is utilized in daily clinical practice in CEE countries. Overall, MSUS is primarily used to monitor RA, while it is utilized as a diagnostic tool in other commonly occurring rheumatologic diseases, such as OA or UA. We found that MSUS was also frequently utilized in the diagnosis of soft tissue disorders; this latter finding likely reflects an unmet diagnostic need. Consequently, MSUS was likely underutilized for other connective tissue diseases, such as systemic lupus erythematosus, in which the diagnosis of musculoskeletal imaging in general plays a smaller role. The number of examined joints was also highest in RA, likely reflecting its polyarticular involvement as compared to the other conditions. The fact that MSUS examination most commonly led to the establishment of a new diagnosis in CPPD deposition disease is in agreement with studies that suggest that MSUS might prove to be a sensitive and specific tool for the diagnosis of CPPD crystal arthritis and may even outperform conventional X-ray imaging [30, 31]. The fact that the percentage of MSUS examinations performed to guide musculoskeletal procedures (i.e., joint and soft tissue injections, arthrocentesis, or biopsies) was relatively lower for inflammatory arthritides (RA, SpA, and UA) and higher for OA and soft tissue disorders might reflect the different role and weight of local interventions within the therapeutic armamentarium for the given conditions.

While we provide novel insights, our study has some limitations. Although nine countries contributed valuable data to the MUST survey, eight additional CEE countries declined or were unable to participate. The overwhelming majority of study centers were university or other hospitals. The extent of possible differences in the routine practices of the individual study sites is also unknown. The MSUS equipment and competence of professionals who participated in the survey also varied considerably between countries and sites. While investigators in the participating centers were requested to document and report all and every MSUS examination conducted within the study period, we cannot rule out sampling bias introduced by the individual investigators. However, given the multinational and multicenter nature of our survey, the influence of such biases, if any, is negligible. Additionally, inherent survey bias may have led to overestimation, in particular with regard to the reasonable use of time and educational value. Also with regard to the latter data and also with regard to the clinical impact of the MSUS examination, our findings are based simply on the physician’s response and may have introduced a certain participation bias, which, however, certainly applies to almost all studies conducted among rheumatologists practicing MSUS. Nevertheless, this does limit the applicability of our results to rheumatologists in general and likely represents the views of rheumatologist experts in MSUS predominantly.

When considering the common diagnoses, which were examined by MSUS, the overall case mix of the study centers should also be taken into account; however, such data were not collected within the framework of the study. Finally, lacking comparable multicenter studies, no reference exists to compare our data with that of other regions of the world.

Overall, we have shown that MSUS may play an important role in the diagnosis and monitoring of commonly occurring rheumatic diseases, as well as on therapeutic decision making in CEE countries. MSUS is considered to be a reasonable use of time and has educational value for both physicians and patients. These results and the differences among the surveyed countries suggest a need to develop a more comprehensive international guideline for the extension of the use of MSUS in view of the current practices in rheumatology.

## Electronic supplementary material

Below is the link to the electronic supplementary material.

Supplementary material 1 (DOCX 51 kb)

## References

[1] Brown AK, Conaghan PG, Karim Z, Quinn MA, Ikeda K, Peterfy CG, Hensor E, Wakefield RJ, O’Connor PJ, Emery P (2008). An explanation for the apparent dissociation between clinical remission and continued structural deterioration in rheumatoid arthritis. Arthritis Rheum.

[2] Brown AK, Quinn MA, Karim Z, Conaghan PG, Peterfy CG, Hensor E, Wakefield RJ, O’Connor PJ, Emery P (2006). Presence of significant synovitis in rheumatoid arthritis patients with disease-modifying anti-rheumatic drug-induced clinical remission: evidence from an imaging study may explain structural progression. Arthritis Rheum.

[3] Balsa A, de Miguel E, Castillo C, Peiteado D, Martín-Mola E (2010). Superiority of SDAI over DAS-28 in assessment of remission in rheumatoid arthritis patients using power Doppler ultrasonography as a gold standard. Rheumatology [Oxford].

[4] Saleem B, Brown AK, Keen H, Nizam S, Freeston J, Wakefield R, Karim Z, Quinn MA, Hensor E, Conaghan PG, Emery P (2011). Should imaging be a component of rheumatoid arthritis remission criteria? A comparison between traditional and modified composite remission scores and imaging assessments. Ann Rheum Dis.

[5] Del Cura JL (2008). Ultrasound-guided therapeutic procedures in the musculoskeletal system. Curr Probl Diagn Radiol.

[6] Mandl P, Naredo E, Conaghan PG, D’Agostino MA, Wakefield RJ, Bachta A, Backhaus M, Hammer HB, Bruyn GA, Damjanov N, Filippucci E, Grassi W, Iagnocco A, Jousse-Joulin S, Kane D, Koski JM, Möller I, De Miguel E, Schmidt WA, Swen WA, Szkudlarek M, Terslev L, Ziswiler HR, Ostergaard M, Balint PV (2012). Practice of ultrasound-guided arthrocentesis and joint injection, including training and implementation, in Europe: results of a survey of experts and scientific societies. Rheumatology (Oxford).

[7] Wakefield RJ, Green MJ, Marzo-Ortega H, Conaghan PG, Gibbon WW, McGonagle D, Proudman S, Emery P (2004). Should oligoarthritis be reclassified? Ultrasound reveals a high prevalence of subclinical disease. Ann Rheum Dis.

[8] D’Agostino MA, Ayral X, Baron G, Ravaud P, Breban M, Dougados M (2005). Impact of ultrasound imaging on local corticosteroid injections of symptomatic ankle, hind-, and mid-foot in chronic inflammatory diseases. Arthritis Rheum.

[9] Naredo E, D’Agostino MA, Conaghan PG, Backhaus M, Balint P, Bruyn GA, Filippucci E, Grassi W, Hammer HB, Iagnocco A, Kane D, Koski JM, Szkudlarek M, Terslev L, Wakefield RJ, Ziswiler HR, Schmidt WA (2010). Current state of musculoskeletal ultrasound training and implementation in Europe: results of a survey of experts and scientific societies. Rheumatology (Oxford).

[10] Naredo E, Bijlsma JW, Conaghan PG, Acebes C, Balint P, Berner-Hammer H, Bruyn GA, Collado P, D’Agostino MA, de Agustin JJ, de Miguel E, Filippucci E, Grassi W, Iagnocco A, Kane D, Koski JM, Manger B, Mayordomo L, Möller I, Moragues C, Rejón E, Szkudlarek M, Terslev L, Uson J, Wakefield RJ, Schmidt WA (2008). Recommendations for the content and conduct of EULAR musculoskeletal ultrasound courses. Ann Rheum Dis.

[11] Terslev L, Hammer HB, Torp Pedersen S, Szkudlarek M, Iagnocco A, D’Agostino MA, Schmidt WA, Uson J, Bruyn GA, Filippucci E, Möller I, Balint P, Wakefield R, Naredo E (2013). EFSUMB minimum training requirements for rheumatologists performing musculoskeletal ultrasound. Ultraschall Med.

[12] Iagnocco A, Porta F, Cuomo G, Delle Sedie A, Filippucci E, Grassi W, Sakellariou G, Epis O, Adinolfi A, Ceccarelli F, De Lucia O, Di Geso L, Di Sabatino V, Gabba A, Gattamelata A, Gutierrez M, Massaro L, Massarotti M, Perricone C, Picerno V, Ravagnani V, Riente L, Scioscia C, Naredo E, Filippou G, Musculoskeletal Ultrasound Study Group of the Italian Society of Rheumatology (2014). The Italian MSUS Study Group recommendations for the format and content of the report and documentation in musculoskeletal ultrasonography in rheumatology. Rheumatology (Oxford).

[13] Kane D, Balint PV, Sturrock R, Grassi W (2004). Musculoskeletal ultrasound—a state of the art review in rheumatology. Part 1: current controversies and issues in the development of musculoskeletal ultrasound in rheumatology. Rheumatology (Oxford).

[14] Wakefield RJ, Goh E, Conaghan PG, Karim Z, Emery P (2003). Musculoskeletal ultrasonography in Europe: results of a rheumatologist-based survey at a EULAR meeting. Rheumatology (Oxford).

[15] Szkudlarek M, Court-Payen M, Strandberg C, Klarlund M, Klausen T, Ostergaard M (2001). Power Doppler ultrasonography for assessment of synovitis in the metacarpophalangeal joints of patients with rheumatoid arthritis: a comparison with dynamic magnetic resonance imaging. Arthritis Rheum.

[16] Weiner SM, Jurenz S, Uhl M, Lange-Nolde A, Warnatz K, Peter HH, Walker UA (2008). Ultrasonography in the assessment of peripheral joint involvement in psoriatic arthritis: a comparison with radiography, MRI and scintigraphy. Clin Rheumatol.

[17] Mandl P, Balint PV, Brault Y, Backhaus M, D’Agostino MA, Grassi W, van der Heijde D, de Miguel E, Wakefield RJ, Logeart I, Dougados M (2012). Metrologic properties of ultrasound versus clinical evaluation of synovitis in rheumatoid arthritis: results of a multicenter, randomized study. Arthritis Rheum.

[18] Mandl P, Naredo E, Wakefield RJ, Conaghan PG, D’Agostino MA (2011). A systematic literature review analysis of ultrasound joint count and scoring systems to assess synovitis in rheumatoid arthritis according to the OMERACT filter. J Rheumatol.

[19] Iagnocco A, Conaghan PG, Aegerter P, Möller I, Bruyn GA, Chary-Valckenaere I, Filippucci E, Gandjbakhch F, Loeuille D, Naredo E, D’Agostino MA (2012). The reliability of musculoskeletal ultrasound in the detection of cartilage abnormalities at the metacarpo-phalangeal joints. Osteoarthr Cartil.

[20] Colebatch AN, Edwards CJ, Ostergaard M, van der Heijde D, Balint PV, D’Agostino MA, Forslind K, Grassi W, Haavardsholm EA, Haugeberg G, Jurik AG, Landewé RB, Naredo E, O’Connor PJ, Ostendorf B, Potocki K, Schmidt WA, Smolen JS, Sokolovic S, Watt I, Conaghan PG (2013). EULAR recommendations for the use of imaging of the joints in the clinical management of rheumatoid arthritis. Ann Rheum Dis.

[21] Mandl P, Navarro-Compán V, Terslev L, Aegerter P, van der Heijde D, D’Agostino MA, Baraliakos X, Pedersen SJ, Jurik AG, Naredo E, Schueller-Weidekamm C, Weber U, Wick MC, Bakker PA, Filippucci E, Conaghan PG, Rudwaleit M, Schett G, Sieper J, Tarp S, Marzo-Ortega H, Østergaard M (2015). EULAR recommendations for the use of imaging in spondyloarthritis in clinical practice. Ann Rheum Dis.

[22] McAlindon T, Kissin E, Nazarian L, Ranganath V, Prakash S, Taylor M (2012). American College of Rheumatology report on reasonable use of musculoskeletal ultrasonography in rheumatology clinical practice. Arthritis Care Res (Hoboken).

[23] Samuels J, Abramson SB, Kaeley GS (2010). The use of musculoskeletal ultrasound by rheumatologists in the United States. Bull NYU Hosp Joint Dis.

[24] Brown AK, Roberts TE, Wakefield RJ, Karim Z, Hensor E, O’Connor PJ (2007). The challenges of integrating ultrasonography into routine rheumatology practice: addressing the needs of clinical rheumatologists. Rheumatology.

[25] Cunnington J, Platt P, Raftery G, Kane D (2007). Attitudes of United Kingdom rheumatologists to musculoskeletal ultrasound practice and training. Ann Rheum Dis.

[26] Iagnocco A, Ceccarelli F, Cuomo G, Delle Sedie A, Filippou G, Filippucci E, Grassi W, Porta F, Sakellariou G (2013). Diffusion and applications of musculoskeletal ultrasound in Italian Rheumatology Units. Reumatismo.

[27] Tamas MM, Fodor D, Rednic N, Rednic S (2011). Musculoskeletal ultrasonography in Romania—results from a specific questionnaire. Med Ultrason.

[28] De Miguel E, Andreu JL, Naredo E, Möller I, Grupo de Trabajo de Ecografía de la Sociedad Española de Reumatología (ECOSER) (2012). Situation of Spanish echography in Spanish rheumatology 2012. Reumatol Clin.

[29] Pineda C, Filippucci E, Chávez-López M, Hernández-Díaz C, Moya C, Ventura L, Grassi W (2008). Ultrasound in rheumatology. The Mexican experience. Clin Exp Rheumatol.

[30] Filippou G, Frediani B, Gallo A, Menza L, Falsetti P, Baldi F, Acciai C, Lorenzini S, Galeazzi M, Marcolongo R (2007). A “new” technique for the diagnosis of chondrocalcinosis of the knee: sensitivity and specificity of high-frequency ultrasonography. Ann Rheum Dis.

[31] Frediani B, Filippou G, Falsetti P, Lorenzini S, Baldi F, Acciai C, Siagkri C, Marotto D, Galeazzi M, Marcolongo R (2005). Diagnosis of calcium pyrophosphate dehydrate crystal deposition disease: ultrasonographic criteria proposed. Ann Rheum Dis.

