# Supplementary material for: Musculoskeletal ultrasonography in routine rheumatology practice: data from Central and Eastern European countries
Source: Rheumatol Int. 2016 Feb 29;36:845–54. doi: 10.1007/s00296-016-3442-2 (PMC4873522; doi:10.1007/s00296-016-3442-2)
Supplement: Supplementary file 1 — Supplementary material 1 (DOCX 51 kb) [file 296_2016_3442_MOESM1_ESM.docx]

**Supplementary Table 1.** **Brand of** **MSUS equipment at study site**

|  | n | % | Transducer frequency range (MHz) |
| --- | --- | --- | --- |
| General Electric | 21 | 47.7 | 4–15 |
| ESAOTE | 17 | 38.6 | 1–18 |
| Philips | 2 | 4.5 | 4–18 |
| Mindray | 2 | 4.5 | 7–10 |
| Other | 3 | 6.8 | 6.5–13 |
| Missing | 1 | 2.3 | - |

n, number of study sites with the respective MSUS equipment; %, percentage of study sites with the respective MSUS equipment; MSUS, musculoskeletal ultrasonography.

Two study sites had more than one MSUS machine available.
